# A Novel *N*-Acetylglutamate Synthase Architecture Revealed by the Crystal Structure of the Bifunctional Enzyme from *Maricaulis maris*

**DOI:** 10.1371/journal.pone.0028825

**Published:** 2011-12-12

**Authors:** Dashuang Shi, Yongdong Li, Juan Cabrera-Luque, Zhongmin Jin, Xiaolin Yu, Gengxiang Zhao, Nantaporn Haskins, Norma M. Allewell, Mendel Tuchman

**Affiliations:** 1 Center for Genetic Medicine Research and Department of Integrative Systems Biology, Children's National Medical Center, The George Washington University, Washington, D. C., United States of America; 2 Key Laboratory of Organo-Pharmaceutical Chemistry, Jiangxi Province, Gannan Normal University, Ganzhou, China; 3 Southeast Regional Collaborative Access Team, Advanced Photon Source, Argonne National Laboratory, Argonne, Illinois, United States of America; 4 Department of Cell Biology and Molecular Genetics and Department of Chemistry and Biochemistry, College of Computer, Mathematical, and Natural Sciences, University of Maryland, College Park, Maryland, United States of America; University of Canterbury, New Zealand

## Abstract

Novel bifunctional *N*-acetylglutamate synthase/kinases (NAGS/K) that catalyze the first two steps of arginine biosynthesis and are homologous to vertebrate *N*-acetylglutamate synthase (NAGS), an essential cofactor-producing enzyme in the urea cycle, were identified in *Maricaulis maris* and several other bacteria. Arginine is an allosteric inhibitor of NAGS but not NAGK activity. The crystal structure of *M. maris* NAGS/K (mmNAGS/K) at 2.7 Å resolution indicates that it is a tetramer, in contrast to the hexameric structure of *Neisseria gonorrhoeae* NAGS. The quaternary structure of crystalline NAGS/K from *Xanthomonas campestris* (xcNAGS/K) is similar, and cross-linking experiments indicate that both mmNAGS/K and xcNAGS are tetramers in solution. Each subunit has an amino acid kinase (AAK) domain, which is likely responsible for *N*-acetylglutamate kinase (NAGK) activity and has a putative arginine binding site, and an *N*-acetyltransferase (NAT) domain that contains the putative NAGS active site. These structures and sequence comparisons suggest that the linker residue 291 may determine whether arginine acts as an allosteric inhibitor or activator in homologous enzymes in microorganisms and vertebrates. In addition, the angle of rotation between AAK and NAT domains varies among crystal forms and subunits within the tetramer. A rotation of 26° is sufficient to close the predicted AcCoA binding site, thus reducing enzymatic activity. Since mmNAGS/K has the highest degree of sequence homology to vertebrate NAGS of NAGS and NAGK enzymes whose structures have been determined, the mmNAGS/K structure was used to develop a structural model of human NAGS that is fully consistent with the functional effects of the 14 missense mutations that were identified in NAGS-deficient patients.

## Introduction

In most microorganisms, fungi, and plants, two different enzymes catalyze the first two steps in arginine biosynthesis, *N*-acetyl-L-glutamate synthase (NAGS, EC 2.3.1.1) and *N*-acetyl-L-glutamate kinase (NAGK, EC 2.7.2.8). However, in *Xanthomonas campestris* and some other bacteria, these reactions are catalyzed by a single bifunctional *N*-acetylglutamate synthase/kinase (NAGS/K), which has been proposed to have evolved from the fusion of ancestral NAGK and *N*-acetyltransferase [1], [2]. In vertebrates, the major physiological role of NAGS seems to be to regulate flux through the urea cycle via activation of carbamyl phosphate synthase by *N*-acetyl-L-glutamate (NAG), the product of the NAGS reaction. Vertebrate NAGS do not have kinase activity, although they retain an amino acid kinase (AAK)-like domain. Vertebrate NAGS have 25–35% sequence identity with bacterial NAGS/K, more than their sequence identity with other bacterial, fungal or plant NAGS, which, like vertebrate NAGS, have a non-functional AAK domain, coupled to the *N*-acetyltransferase domain (NAT).

Many NAGS, NAGK, and NAGS/K enzymes are allosterically regulated by L-arginine. In organisms that have a linear arginine biosynthetic pathway such as *Escherichia coli*, the target of arginine feedback inhibition is NAGS. In organisms that have a cyclic pathway such as *Pseudomonas aeruginosa*, the main target of feedback inhibition is NAGK [3]. However, in terrestrial tetrapods, arginine is an activator of NAGS. The transition from inhibition to activation appears to have occurred when tetrapods migrated from sea to land and needed a robust system for eliminating ammonia [4]. In the only NAGS crystal structure that has been determined, that from *Neisseria gonorrhoeae* (ngNAGS), the arginine binding site is located in the AAK-like domain, and arginine binding is accompanied by substantial structural changes [5].

Although the crystal structures of several NAGK enzymes have been determined [6], [7], [8], [9], NAGS enzymes have proven more challenging and the only NAGS structure that has been determined is that of *Neisseria gonorrhoeae* [10]. This enzyme is a member of the classical bacterial and plant NAGS family, and has less than 18% sequence similarity to mammalian NAGS enzymes. We have now succeeded in solving the structures of two NAGS/K enzymes, from *Maricaulus maris* (mmNAGS/K) and *X. campestris* (xcNAGS/K) that have substantially higher sequence similarity to vertebrate NAGS. In both crystals and solution, ngNAGS is consistently hexameric, while mmNAGS/K and xcNAGS/K, as determined herein, are tetrameric. Thus these new structures are of interest in terms of understanding the evolution and mechanisms of NAGK, NAGS, and NAGS/K enzymes, providing potential insights into human NAGS, and perhaps in developing a strategy for determining the crystal structure of the human enzyme.

## Results

### Enzymatic activity

**Figure 1:**
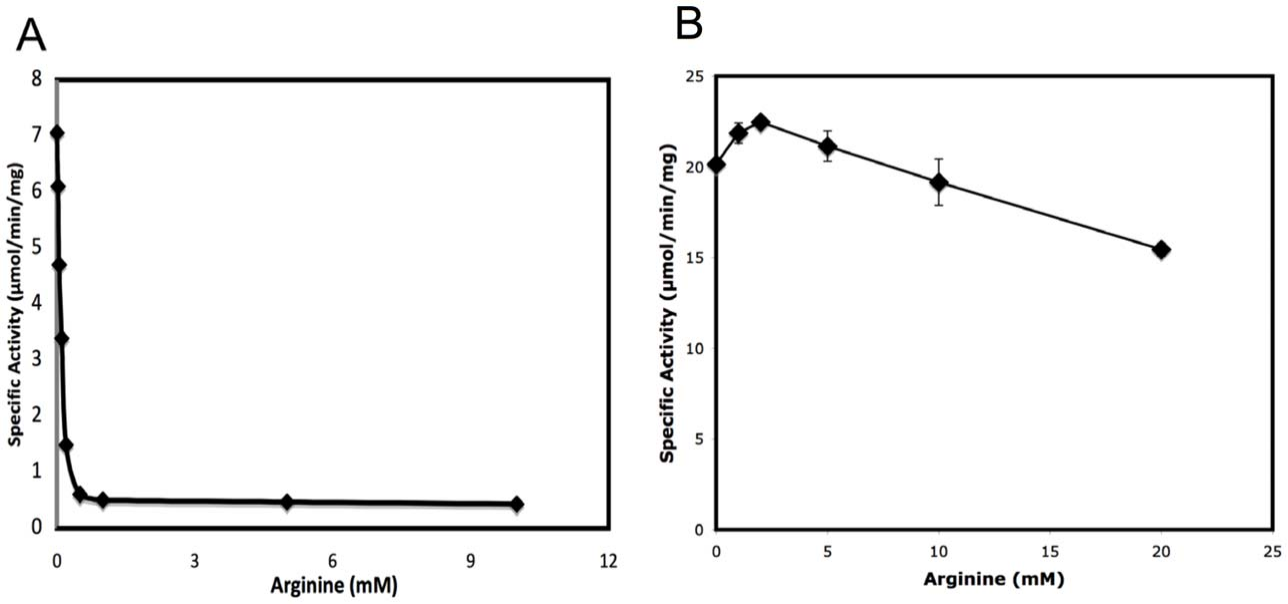
Effect of L-arginine on the NAGS and NAGK activity of mmNAGS/K. A. NAGS specific activity (µmol/min/mg) was measured at 2.5 mM AcCoA and 10 mM L-glutamate in a buffer containing 50 mM Tris-HCl pH 8.5 and a range of L-arginine concentrations (0, 0.02, 0.05, 0.1, 0.2, 0.5, 1, 5 and 10 mM). B. NAGK specific activity (µmol/min/mg) was measured at 20 mM ATP and 100 mM NAG in a buffer containing 100 mM NaCl, 40 mM MgCl_2_, 400 mM hydroxylamine, 20 mM Tris-HCl pH 7.4 and a range of concentrations of L-arginine (0, 1, 2, 4, 10 and 20 mM). Reactions were performed at 310 K for 30 min.

We have previously shown that xcNAGS/K has both NAGS and NAGK activity and that its NAGS activity, but not NAGK activity, is inhibited completely by 1 mM L-arginine [1]. As shown in Figure 1, we demonstrate here that mmNAGS/K has both NAGS (7.0 µmole/min/mg) and kinase activity (20.1 µmole/min/mg) and that its NAGS activity is inhibited 14 fold by 1.0 mM L-arginine. NAGK activity is not inhibited by L-arginine at physiological concentrations; instead, slight activation is observed at 1 mM L-arginine. 76% of the maximal kinase activity is retained even at 20 mM of L-arginine. These results are consistent with those reported previously for xcNAGS/K [1], which has 7.0 µmole/min/mg kinase activity at pH 6.0 and is only slightly inhibited by 1 mM L-arginine. The kinase activity of mmNAGS/K is somewhat lower than that of *Thermotoga maritima* NAGK (tmNAGK) (45 µmole/min/mg), *P. aeruginosa* NAGK (130 µmole/min/mg) and *E. coli* NAGK (ecNAGK) (64 µmole/min/mg) [11], [12], perhaps because of different assay conditions. Arginine appears to regulate arginine biosynthesis in both *X. campestris* and *M. maris* mainly by inhibiting their NAGS activity, as is the case for *E. coli* [3]. This pattern may be characteristic of bacteria that have a linear arginine biosynthetic pathway, and do not have an ornithine *N*-acetyltransferase gene.

### Cross-linking experiments

**Figure 2:**
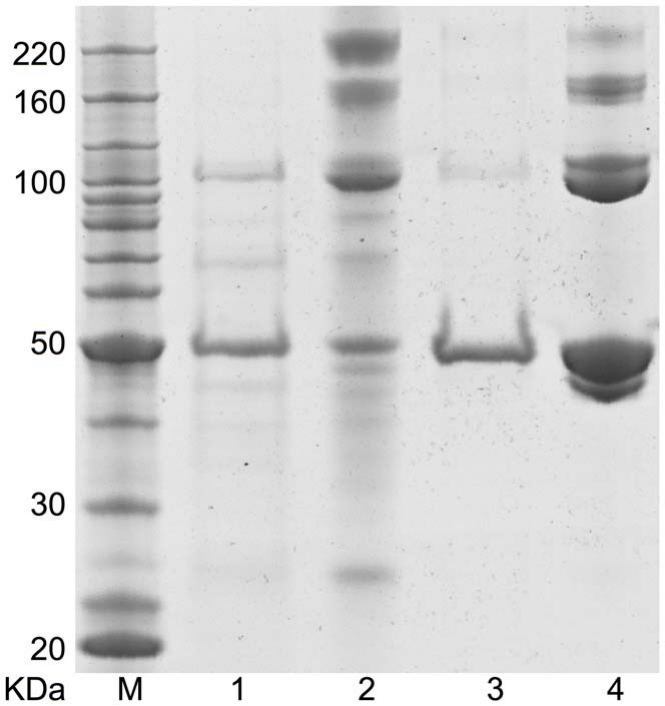
Cross-linking of recombinant xcNAGS/K and mmNAGS/K proteins with dimethyl suberimidate. mmNAGS/K and xcNAGS/K (0.15 µg) were incubated with dimethyl suberimidate (0.25 µg) in 50 µl solution containing 200 mM triethanolamine, pH 8.25 for three hours at 298 K. Lanes M, protein size markers; 1, xcNAGS/K without cross-linking reagent; 2, xcNAGS/K with cross-linking reagent; 3, mmNAGS/K without cross-linking reagent; 4, mmNAGS/K with cross-linking reagent.

To determine the state of oligomerization of both xcNAGS/K and mmNAGS/K in solution, cross-linking experiments were performed with dimethyl suberimidate as the cross-linking agent. Four major bands were seen for both enzymes with SDS-PAGE, with molecular weights corresponding to oligomers of 1, 2, 3 and 4 subunits (Figure 2, Lane 3 and 4). Thus, xcNAGS/K and mmNAGS/K appear to exist primarily as tetramers in solution.

### mmNAGS/K structure determination

Determining the structure of a bifunctional NAGS/K initially proved challenging. Of nine NAGS/K and NAGS/K-like genes that we cloned (*X. campestris*, *X. fastidiosa*, *M. maris*, *O. alexandrii*, *S. maltophilia*, *P. bermudensis*, *S. cellulosum*, *K. koreensis* and *H. ochraceum*), only NAGS/K from *X. campestris* and *M. maris* produced diffracting crystals.

As noted in Methods, crystals of mmNAGS/K were obtained in trigonal space group P3_1_21, hexagonal space group P6_2_22 and orthorhombic space group P2_1_2_1_2_1_. Crystals of the first two forms diffracted poorly and were difficult to reproduce. Although the last crystal form was also difficult to reproduce, crystals that diffracted to ∼3.0 Å were obtained (Table 1). Several MAD data sets collected from Se-Met substituted wild-type protein were not of sufficient quality to locate the selenium positions. To increase phasing power, three additional amino acids codons were mutated to methionine (I106M, I294M and L367M). Crystals from this mutant protein diffracted to 2.7 Å (Table 1) and phase information was obtained from the MAD dataset. The asymmetric unit was found to consist of four subunits assembled as a tetramer, and unit cell parameters are consistent with four subunits per asymmetric units and 50% solvent content [13].

**Table 1:** Diffraction data and refinement statistics for native protein and Se-MAD data.

	Native	Se-edge	Se-inflection	Se-remote
Data				
Space group	P2_1_2_1_2_1_	P2_1_2_1_2_1_	P2_1_2_1_2_1_	P2_1_2_1_2_1_
Wavelength (Å)	1.0000	0.97921	*0.97934*	0.97471
Resolution (Å)	40-3.1	40.0-2.67	*40.0-2.8*	40.0-2.66
Highest resolution shell (Å)	3.2-3.1	2.73-2.67	2.87-2.80	2.73-2.66
Unit-cell parameters (Å)	*a* = 114.6	*a* = 113.3	*a* = 113.5	*a* = 113.4
	*b* = 118.2	*b* = 117.4	*b* = 117.6	*b* = 117.5
	*c* = 152.3	*c* = 149.1	*c* = 149.4	*c* = 149.3
Measurements	251,381	408,041	366,144	413,198
Unique reflections	36,398 (2,838)	56,638 (3,630)	50,373 (3,290)	57,212 (3,679)
Redundancy	6.9 (5.8)	7.2 (5.1)	7.3 (6.2)	7.2 (5.8)
Completeness (%)	95.5 (76.2)^a^	99.8 (98.0)	99.8 (99.8)	99.8 (98.1)
*R*_merg_ b	0.092 (0.607)	0.092 (0.748)	0.096 (0.920)	0.072 (0.577)
<*I/σ*(*I*)>	21.7 (2.1)	27.3 (1.6)	26.3 (1.7)	36.8 (2.6)
Refinement				
Reflections, working set	34,446 (2,248)			104,278 (8.580)
Reflections, test set	1,909 (108)			3,677 (333)
Total atoms (non-H)	13,265			13298
Protein atoms	12,159			13144
Ligands				
CoA	2			1
Glutamate	2			0
Malonate	0			2
Others	0			3
Waters	6			84
*R*	0.189 (0.254)			0.183 (0.276)
*R*_free_	0.306 (0.359)			0.256 (0.325)
Rmsd bond lengths (Å)	0.009			0.009
Rmsd bond angles (°)	1.288			1.210
Average B factor (Å^2^)				
Protein	118.3			82.1
CoA	170.0			121.9
Glutamate	93.0			
Water	58.3			55.2
Ramachandran plot (%)				
Favored	75.5			85.5
Allowed	22.2			12.9
Generous	1.7			1.8
Disallowed	0.6			0.1

a Figures in brackets apply to the highest-resolution shell.

b *R*_merg_ = Σ*_h_*Σ*_i_*|*I*(*h*,*i*)-<*I*(*h*)>|/Σ*_h_*Σ*_i_I*(*h*,*i*), where *I*(*h*,*i*) is the intensity of the *i*th observation of reflection *h*, and <*I*(*h*)> is the average intensity of redundant measurements of reflection *h*.

Cell parameters for the trigonal crystal form of mmNAGS/K are *a* = *b* = 95.1, *c* = 253.0 Å and β = 120°, consistent with two subunits per asymmetric unit and 42% solvent content. Even though this crystal form diffracts to only 4.3 Å resolution, the structural solution in space group P3_1_21 could be found by molecular replacement using the A–X dimer of the P2_1_2_1_2_1_ crystal form as the search model. Packing analysis indicates that the 2-fold symmetry axis of the molecular tetramer is aligned with the 2-fold crystallographic axis perpendicular to the plane of the tetramer ring.

### xcNAGS/K structure determination

Crystals of xcNAGS/K diffracted anisotropically and had high solvent content (75%) [2]. MAD data collected from these crystals allowed the selenium sites to be identified, but did not have sufficient resolution for reliable model building, particularly of the NAT domain, and subsequent model refinement. However, the low resolution electron density suggested that there is only one subunit in the asymmetric unit, and that four subunits assemble to form a tetramer aligned with crystallographic symmetries, so that the xcNAGS/K tetramer has exact P222 point symmetry. The molecular replacement solution with the mmNAGS/K subunit structure as the search model confirmed these conclusions and assignment to space group P6_2_22 (Table S1).

Since the cell parameters of the hexagonal crystal form of mmNAGS/K are similar to those of xcNAGS/K crystals, this structure would be expected to be isomorphous to the xcNAGS/K structure, with only one subunit in the asymmetric unit.

### Structural variation within the tetramer

Within the P2_1_2_1_2_1_ mmNAGS/K tetramer, there are significant differences between the subunits, indicating that they have sufficient innate flexibility to respond to different packing environments. The RMSD for superposition of the four subunits is 1.5–2.0 Å, and subunits X and A are better defined than subunits Y and B. The resolution of the electron density of several loop regions in subunit Y, such as H4–H5, B9–B10 and H8–H9, does not allow their structures to be modeled. The RMSD value decreases to 0.8–1.2 Å when only AAK domains are superimposed and to 0.4–0.7 Å if only NAT domains are superimposed, indicating that the AAK domain has more structural flexibility than the NAT domain. The RMSD value decreases further to 0.2–0.5 Å if only the core β-sheets are superimposed for both NAT and AAK domains, demonstrating conservation of these core structures. Structural variation among subunits in an asymmetric unit has been observed in *P. aeruginosa* NAGK, where the average RMSD was as high as 2.1 Å when all Cα atoms in the 12 subunits in the asymmetric unit were superimposed [8].

The structural variation between equivalent subunits in the native protein and Se-Met substituted mutant is much lower than between subunits within a tetramer. The RMSD between equivalent subunits is 0.4–0.5 Å for subunits A, B, and X, and 0.8 Å for subunit Y.

### Structures of AAK and NAT domains

**Figure 3:**
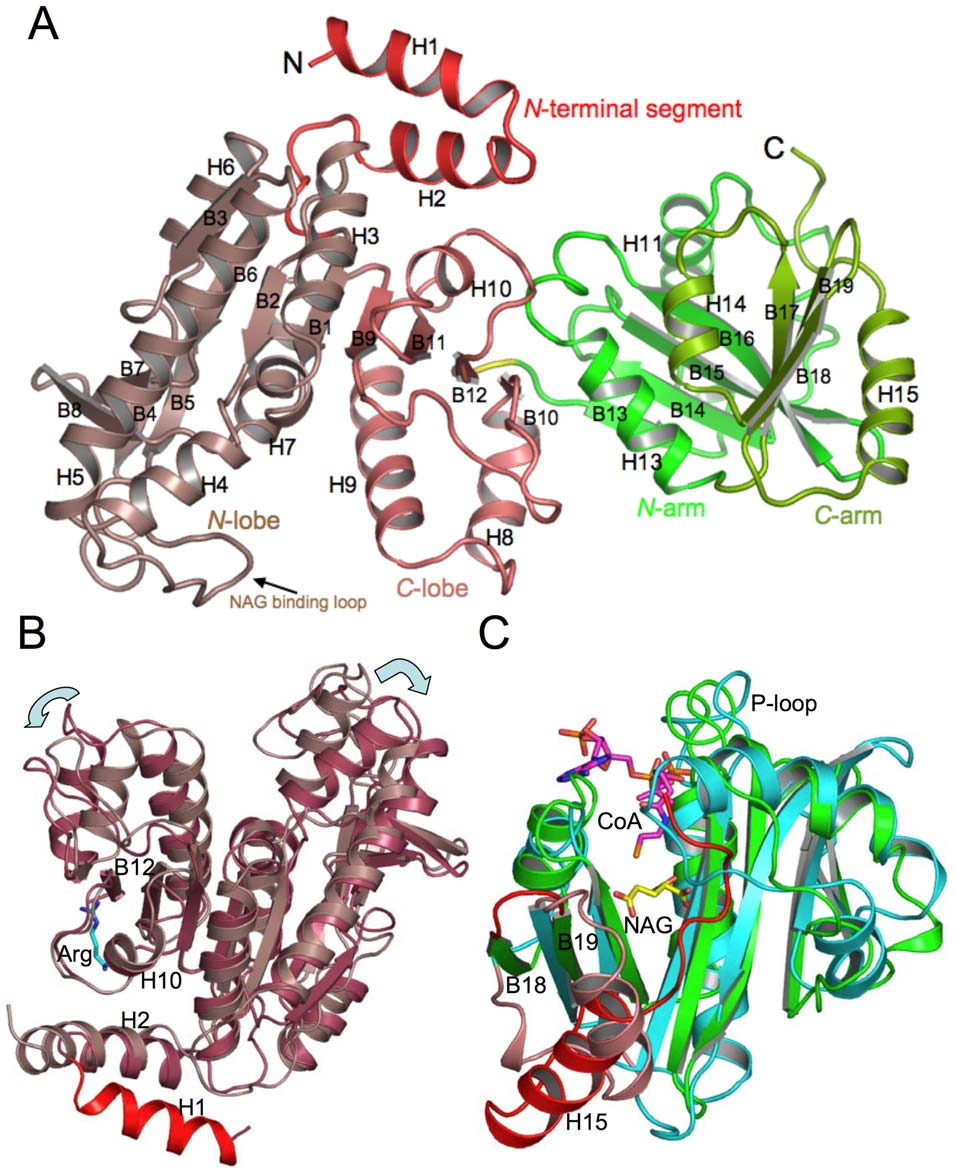
Ribbon diagrams of the subunit structure of mmNAGS/K. A. Structural elements within the subunit are shown in different colors: red, *N*-terminal segment; brown, *N*-terminal lobe of AAK domain; pink, *C*-terminal lobe of AAK domain; dark green, *N*-terminal arm of NAT domain; light green, *C*-terminal arm of NAT domain. Gly291, the linker between the AAK and NAT domains, is shown in yellow. B. Superimposition of the AAK domains of mmNAGS/K (brown) and ngNAGS (reddish brown). Arginine bound to ngNAGS is shown as blue sticks, and the extra *N*-terminal helix of mmNAGS/K is shown in red. Note that the AAK domain of mmNAGS/K is in an open conformation (indicated by arrows) relative to ngNAGS. C. Superimposition of NAT domains of mmNAGS/K (dark green) and ngNAGS (blue). Regions that differ significantly in the two proteins are shown in red (mmNAGS/K) and salmon (ngNAGS), respectively. CoA bound between the *N*-terminal and *C*-terminal arms of ngNAGS is shown as pink sticks. The bound NAG in ngNAGS is shown as yellow sticks.

As shown in Figure 3A, each mmNAGS/K subunit has two domains, the AAK domain (residues 1–290) and the NAT domain (residues 292–441) connected by a hinge residue, Gly291.

The AAK domain has the typical AAK fold seen in other NAGK structures, with an eight- strand twisted β-sheet core (arranged as B3↓, B6↓, B2↓, B1↓, B9↓, B11↓, B12↑, B10↑), with three α helices (H5, H3 and H10) on one side and four α helices (H6, H7, H9 and H8) on the other, forming a α_3_β_8_α_4_ sandwich structure (Figure 3A). α-helix H4 extends from the C-edge of β strand B2. A long loop connecting H4 and H5 hangs over the C-edge of the β-sheet core and contains a putative NAG binding residue, Arg99, equivalent to Arg66 in ecNAGK. A small β-sheet consisting of four short β strands (arranged as B8↑, B7↓, B4↓, B5↑) protrudes from the C-edge of the core and forms part of the flat dimerization interface with H5, B3 and H6.

Structural homology searches using the DALI server [14] indicate that the AAK domain is most similar to NAGK from *T. maritima* (PDB: 2BTY, Z = 28.2, RMSD = 2.7 Å) with over 262 aligned residues and 26% sequence identity. The AAK domain from ngNAGS is the second best match (PDB: 2BTY, Z = 27.7, RMSD = 2.7 Å) with over 260 aligned residues and 21% sequence identity.

The AAK domain can be divided further into three regions: *N*-terminal segment (residues 1–39), *N*-terminal lobe (40–204) and *C*-terminal lobe (205–290) (Figure 3A). The *N*-terminal segment has two helices, in contrast to arginine sensitive NAGK and ngNAGS, both of which have only one mobile *N*-terminal helix (Figure 3B) [8], [10], and arginine insensitive NAGK which has no *N*-terminal segment [9].

The NAT domain has a characteristic GCN5-related *N*-acetyltransferase (GNAT) fold with a central twisted seven-strand β-sheet surrounded by six α helices (Figure 3A). The central β-sheet consists of a four-strand anti-parallel sheet (*N*-terminal arm, residues 292–380, arranged as B13↓, B14↑, B15↓, B16↑) and a three-strand anti-parallel sheet (*C*-terminal arm, residues 381–441, arranged as B17↑, B19↓, B18↑). These two sheets form a V-shaped structure, with adjacent β-strands (B16 and B17) parallel. V-shaped central β-sheets are characteristic of the GNAT family and are believed to be essential for catalysis of the acetyl transferase reaction [15], [16], [17]. The best match with the NAGK/S NAT domain found in a structural neighbor search is the GNAT protein that catalyzes acetylation of the ribosomal protein S18 (PDB 2cnm; Z = 13.2, RMSD = 2.4 Å) with over 123 aligned residues and 19% sequence identity [18]. GNAT proteins have highly variable sequences, probably because bound AcCoA interacts primarily with backbone rather than side-chain atoms, and, as a result, the 7^th^ best match (PDB 3ne7; Z = 12.0, RMSD = 2.4 Å) with over 119 aligned sequences, has only 6% sequence identity.

Although the NAT domain from ngNAGS is not in the top 50 matches, superimposition of the NAT domain from mmNAGS/K with that of ngNAGS resulted in an RMSD of 2.5 Å with 112 aligned residues and 15.2% sequence identity (Figure 3C). The V-shaped core structure of the central β-sheet in the NAT domain is similar in both structures [10]. However, there are significant differences, particularly in the *C*-terminal arm. The loop that links β-strands B18 and B19 is much shorter in mmNAGS/K than in ngNAGS, which has two extra helices (H14′ and H14). Instead, the structure of mmNAGS/K has a long H15 α-helix, occupying the position equivalent to H14 of ngNAGS and replacing its B24–B25 linker. Since this linker contributes Arg425 and Ser427 to the glutamate binding site of ngNAGS, mmNAGS/K must bind glutamate with other residues.

### Tetramer structure

**Figure 4:**
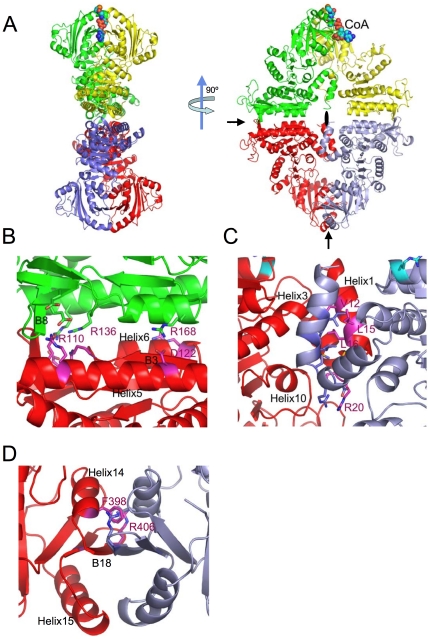
Structure of the mmNAGS/K tetramer and interfaces between subunits shown as ribbon diagrams. A. The tetramer is shown in two different orientations, perpendicular to the plane of the ring, and parallel to the plane of the ring. Subunit A (red), subunit B (green), subunit X (purple -gray) and subunit Y (yellow). Bound CoA molecule is shown as a space-filling model. The two 2-fold non-crystallographic rotation axes in the plane of the ring are indicated by arrows and the 2-fold non-crystallographic rotation axis perpendicular to the plane of the ring is indicated by a filled oval. B. ecNAGK-like AAK-AAK domain interface between subunits A and B; α-helices, H5 and H6, and β-strands, B3 and B8, form this interface. C. *N*-terminal helix interface between subunits A and X, formed by interactions between the two *N*-terminal α-helices and two neighboring helices, H3 and H10. D. NAT-NAT domain interface between subunits A and X. Two α-helices, H14 and H15, and one β-strand, B18, form this interface.

The four subunits of the mmNAGS/K tetramer form an elongated 35–74 Å thick ring with a long axis of 140 Å and a short axis of 108 Å (Figure 4A). The tetrameric structures of the other two crystal forms (space groups: P6_2_22 and P3_1_21) are similar (Figure S1A). Since the same tetramer is formed regardless of crystal conditions and crystal packing, it would be expected to be the predominant form in solution, and cross-linking experiments confirm that xcNAGS/K and mmNAGS/K exist as tetramers in solution (Figure 2).

Interface interactions between subunits are extensive and of three types. There are two types of interactions between AAK domains—interactions between adjacent subunits (A–B or X–Y) within the ring, and interactions between subunits located on opposite sides of the ring (A–X or B–Y). In addition, there are interactions between the NAT domains of opposite subunits (A–X or B–Y).

The first AAK-AAK interface, between adjacent subunits in the ring, is similar to the dimerization surface of ecNAGK and other enzymes of the AAK family [19] (Figure 4B). However, the specific interactions are unique to mmNAGS/K, and different from those of any dimer interface previously reported for any NAGK or any other members of the AAK family, such as carbamate kinase, glutamate 5-kinase and UMP kinase [19], [20], [21]. Here, the interface consists of β strand B3 and two α helices, H5 and H6, arranged as an αβα sandwich. The interacting α-helices, H5 and H6, (equivalent helices αC and αD in ecNAGK), and β-strand, B3, are almost parallel to the equivalent elements from the adjacent subunit. Residues from H6 interact with those from H5 of the adjacent subunit via numerous hydrophobic and hydrogen bonds. Since the distance between the two β-strands (B3) from adjacent subunits is about 5.0 Å, their backbone atoms cannot hydrogen bond directly. Instead, a small β-strand, B8, interacts with the equivalent element from the adjacent AAK domain in an anti-parallel mode to form an interface unique to mmNAGS/K (Figure 4B). In total, approximately 20 hydrogen bonds, three pairs of salt bridges (Arg168-Asp122, Arg110-Glu140, and Arg136-Glu180) and numerous hydrophobic interactions are involved in forming this interface.

This interface is flat with a buried area of 942 Å^2^, calculated using PISA server [22] and a probe radius of 1.4 Å, significantly less than those in tmNAGK (1381 Å^2^) and ecNAGK (1279 Å^2^).

The second interface, between the AAK domains of subunits on opposite sides of the ring, involves interactions between parallel helices of the *N*-terminal segment (Figure 4C), analogous to the interactions of *N*-terminal helices of the AAK domains of ngNAGS and ecNAGK; however, the specific interactions are different. The calculated inaccessible surface of 968 Å^2^ for the A–X subunit interface is larger than that of 555 Å^2^ for the B–Y subunit interface. The smaller value for the latter interface may reflect the distinctive conformation of the subunit Y, and/or the increased disorder of its *N*-terminal helices relative to the other three subunits, which led to fewer residues being included in the structural model. The residues involved in this interface interaction are mainly hydrophobic and include Ile12, Leu15, Leu16, Met19 and Phe63, as well as one salt bridge between Arg20 and Asp21 of opposing subunits and two hydrogen bonding interactions, between Met19 O and Arg276 NH2, and between His18 NE2 and Ser59 OG. In contrast, in ngNAGS and arginine sensitive NAGK structures, the *N*-terminal helices interlace with adjacent AAK domains to form hexameric structures [6], [7], [8], [10].

At the third interface, the *C*-terminal arms of NAT domains of opposite subunits (A–X and B–Y) form a continuous 6-strand anti-parallel β-sheet (Figure 4D). This extensive interface has a buried area of 1485 Å^2^. The primary interactions consist of backbone hydrogen bonding between equivalent B18 β-strands and cation-π-π-cation stacking interactions between alternating side chains of Phe398 and Arg406 from opposite subunits. Similar interfaces are found in other enzymes with GNAT folds that form dimers in solution using their *C*-terminal arms as an interface [15], [17]. In contrast, no NAT-NAT domain interactions were identified in the ngNAGS structure [10].

### The putative NAGK active site and arginine binding site in the AAK domain

Superimposition of the AAK domain of the mmNAGS/K structure with the ecNAGK (PDB 1gs5) structure [9] strongly suggests that the substrate binding sites and catalytic mechanism of their NAGK reactions are very similar. Active site residues K8, D181 and K217 that interact with the phosphate groups of ATP in ecNAGK are also found in mmNAGS/K (K44, D192 and K249, respectively) and are located in similar positions (Figure S2A). Active site residues Gly185 and Gly213 which are part of the ATP binding pocket in ecNAGK, are also conserved (equivalent residues Gly215 and Gly245 in mmNAGS/K). Other important residues such as Gly11 and Gly44 (equivalent residues Gly47 and Gly77 in mmNAGS/K), whose backbone nitrogen atoms hydrogen bond to the γ-phosphate group of ATP or the phosphate group of NAG phosphate in ecNAGK, are conserved as well. Key residues involved in binding NAG are also conserved (G64, R66 and N158 in ecNAGK, equivalent residues G97, R99 and N190 in mmNAGS/K) (Figure S2A).

**Figure 5:**
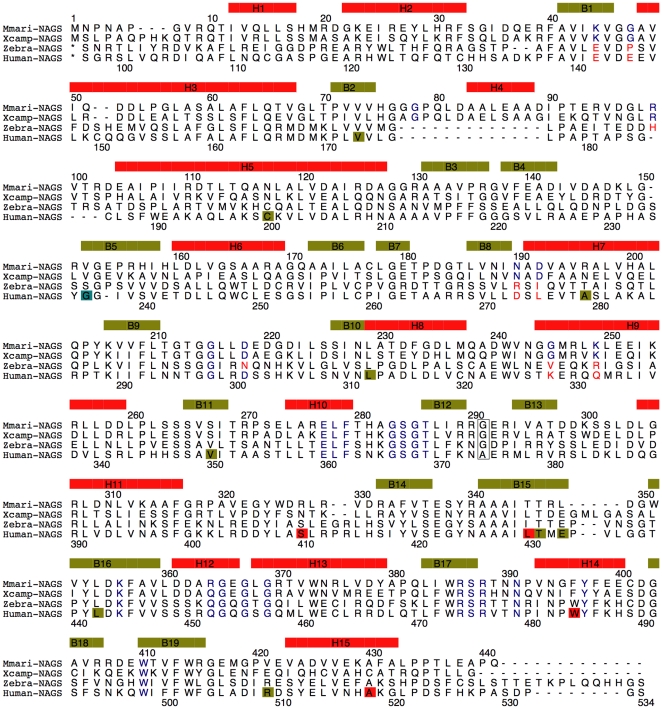
Sequence alignment of mmNAGS/K, xcNAGS/K, zebra fish NAGS and human NAGS. The sequence encoding secondary structure elements are indicated by boxes in yellow-green (β-strand) and red (α-helix). Encoded amino acids that are modeled in binding of ligands (ATP, NAG, AcCoA, glutamate, L-arginine), are indicated in blue. The linker residue, Gly291, is boxed. The missense human mutations are indicated by boxes in red, yellow-green, light blue for neonatal, late-onset and unknown onset patients, respectively.

The characteristic sequence motif of the arginine binding site, E-L-F-(T/S)-x-x-G-x-G-T, is strongly conserved across arginine sensitive bacterial NAGK, fungal NAGK, classic bacterial NAGS, bifunctional NAGS/K, and vertebrate NAGS [1], [23] (Figure 5). The structure of this binding site is also conserved in arginine sensitive NAGK and ngNAGS [5], [8]. The corresponding site in mmNAGS/K consists of the cavity formed by the loop connecting helix H10 and β strand B12 (residues 277–287) (Figure S2B). In tmNAGK, *Arabidopsis thaliana* NAGK (atNAGK), and ngNAGS structures, residues in the *N*-terminal helix (H1) (Tyr15 in tmNAGK, Lys32 and Phe33 in atNAGK, Tyr17 in ngNAGS) form part of arginine binding site [5], [7], [8]. In mmNAGS/K, Tyr28 and nearby residues in the second *N*-terminal helix, H2, are positioned to play this role.

The mechanisms by which arginine binding strengthens interdomain interactions in ngNAGS and mmNAGS/K may also be similar. In ngNAGS, arginine binding enhances AAK-NAT domain interactions by creating new hydrogen bonding interactions between Arg255-Asp334, Tyr17-Asn336 and Arg274-Gln362 [5]. The arginine binding site in mmNAGS/K is close to the NAT domain “P-loop” which is likely to interact with the pyrophosphate group of AcCoA. This creates the possibility of bound arginine or nearby residues in the AAK domain interacting with residues such as Glu366 in the “P loop”, thereby regulating the binding of AcCoA.

Since mutations in the arginine binding site of the kinase domain of both mouse NAGS and xcNAGS/K [4] eliminate arginine's effect on NAGS activity, bifunctional NAGS/K would not be expected to have two arginine binding sites, one affecting NAGS activity and the other one affecting NAGK activity. Although arginine has been reported to inhibit the NAGS activity of the short version of NAGS, which consists of only the NAT domain, from *Mycobacterium tuberculosis* [24], it has not yet been shown that this NAGS domain has a specific arginine binding site.

### Non-productive and putative CoA binding sites

**Figure 6:**
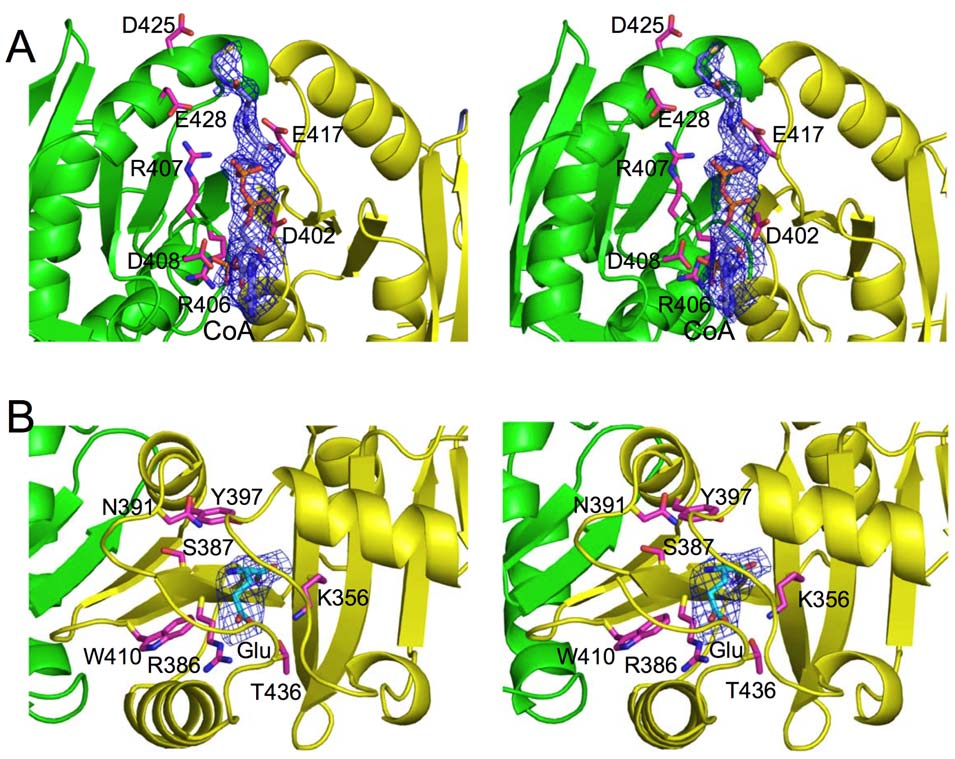
CoA and glutamate binding sites of mmNAGS/K. Bound CoA and glutamate molecules are shown as pink sticks. Omit electron density maps (Fo-Fc) around bound CoA (contoured at 2.0 σ) and glutamate (contoured at 3.0 σ) are shown as blue cages. The side chains of surrounding residues are shown as pink sticks. A. The non-functional CoA binding site in the cleft formed by adjacent NAT domains. Residues Arg406, Arg407, Glu408, Asp425 and Glu428 from subunit B, and Asp402, Trp414, Glu417 from subunit Y form this CoA binding cleft. The sulfur atom of CoA is fully exposed to the solvent. B. Glutamate binding site in the native mmNAGS/K structure, remote from the non-functional CoA binding site.

Although both native and mutant mmNAGS/K were incubated with 25 mM CoA before crystallization, only one bound CoA molecule per tetramer, with incomplete occupancy, was identified in the cleft between the NAT domains of subunits B and Y in the mutant structure (Figure 6A). In the native structure, this CoA molecule was visible, as well as a second molecule in the cleft between subunits A and X (Figure S1B). This CoA binding site does not correspond to the CoA binding site in ngNAGS [10] and other GNAT enzymes [17], [25] and is unlikely to be functional, since most of the residues forming the binding site are not conserved and there is no glutamate binding site close by. The sub-optimal pH and high ionic strength of the crystallization conditions may be a factor in CoA binding to a non-functional site rather than the native active site. Substrate binding at a non-functional site rather than the active site has been observed in many other enzymes [26], [27].

Although sequence similarity among GCN5-fold acetyltransferases is low (6–12% sequence identity), the AcCoA binding site is conserved across all enzymes that have been studied [25], [28]. Therefore, the protein residues that interact with AcCoA in mmNAGS/K can be predicted by superimposing other known acetyltransferase structures [5], [10], [18] (Figure 3C and Figure S2C). The “P-loop” (Arg364-Gly365-Glu366-Gly367-Leu368-Gly369) is consistent with the consensus sequences [Q/R]-x-x-G-x-[G/A] [29] characteristic of AcCoA binding sites and is located close to the pyrophosphate moiety. The S-acetylpantetheine moiety of AcCoA probably forms a pseudo-antiparallel β-sheet interaction with B16, positioning the acetyl group in almost the same plane as the β-sheet. Tyr397 and Ser387 are within hydrogen bonding distance of the sulfhydryl group of AcCoA and could act as the active site acid and base, respectively. A highly conserved tyrosine, which is equivalent to Tyr397 in mmNAS/K, has been identified in several other GCN-5 related protein structures and has been considered to be catalytically important [28].

### Glutamate binding site

Although the sequence similarity between mmNAGS/K and ngNAGS is too low to locate the glutamate binding site, structure alignment enables the probable site to be identified. In all known acetyltransferase structures, the substrate that accepts the acetyl group from AcCoA approaches from the opposite side of the β-sheet (the same side as α-helix H15) for in-line nucleophilic attack on the *Re* face of the acetyl group of AcCoA. Therefore, the glutamate site in mmNAGS/K is likely to correspond to this site in ngNAGS (PDB 3d2m) [10]. In the native mmNAGS/K structure, there is residual electron density in subunits A and Y at this site that can be modeled as L-glutamate (Figure 6B) suggesting that this site is likely to be the biologically relevant L-glutamate binding site. The equivalent site in the Se-Met substituted mutant structure has residual electron density that can be modeled as malonate which was present at a high concentration in the crystallization buffer.

The γ-carboxyl group of L-glutamate appears likely to be anchored by Arg386, Lys356 and Thr436 (Figure 6B), while the α-carboxyl group may be fixed by hydrogen bonding interactions with the main chain N atom of Phe357 and the main chain O atom of Arg386. The side chains of hydrophobic residues Phe316, Leu437 and Trp410 are also part of the L-glutamate binding site. The residues involved in binding L-glutamate are highly conserved. Lys356 and Phe357 are part of the conserved motif Tyr353-Leu354-Asp355-Lys356-Phe357 (YLDKF), and Arg386 is part of the conserved motif Trp385-Arg386-Ser387-Arg388 (WRSR). These conserved motifs are found in all bifunctional NAGS/K, as well as vertebrate and fungal NAGS, implying a common binding mechanism (Figure 5). Similarly, Tyr397, Ser387 and Asn391, which have roles in the catalytic reaction, are also highly conserved, implying a common catalytic mechanism.

### Protein flexibility encoded intrinsically in the structure

The variability in the structures of the four subunits in the asymmetric unit of the P2_1_2_1_2_1_ crystal and in crystals obtained under different conditions and in different space groups provides an opportunity to study the conformational range of NAGS/K and its relationship to the catalytic and regulatory mechanisms. When the β-sheet cores of the AAK domain of the Se-Met substituted mutant are superimposed, it is immediately apparent that the NAT domain can adopt different orientations relative to the AAK domain (Figure S3A). Relative to subunit Y, the NAT domains of subunits B, X, and A are rotated 25.2°, 24.7°, and 16.9° toward the AAK domains, respectively. To test whether the difference in relative domain orientation in subunits Y and B might be related to L-arginine binding, CoA and L-arginine were modeled in their proposed binding sites. The clefts between the AAK and NAT domains of subunits B and X are in a closed conformation, creating a steric clash between bound CoA and the arginine binding loop (residues 281–287) (Figure S3B). This clash does not exist in subunit Y (Figure S3C). The closed conformation of subunits B and X may represent the conformation that exists when L-arginine is bound, while the open conformation of subunit Y and probably subunit A may represent the active form without L-arginine bound. Interestingly, in native mmNAGS/K, glutamate can only be identified in subunit Y and A (Figure S1B).

There may also differences in the inter-lobe movements of the AAK domains of different mmNAGS/K subunits and between mmNAGS/K and xcNAGS/K. AAK inter-lobe movement has been demonstrated in ecNAGK [30]; upon ATP binding the *C*-terminal lobe rotates 24°–28° towards the *N*-terminal lobe, where the NAG binding site is located. The conformations of the AAK domain in our structures are all in the open conformation, which is consistent with structures without ATP or ADP bound.

In addition to the large inter-domain rotation between the AAK and NAT domain, and inter-lobe movement within the AAK domain, several loops in the AAK domain may exhibit large movements. Specifically, the NAG binding loop (residues 89–104), which contains a NAG binding residue, Arg99 (Figure S2A), would be expected to move more than 5.0 Å when NAG binds. In the mmNAGS/K structure, the NAG binding loop is very flexible, reflected by a weak electron density.

In contrast to the AAK domain, the NAT domain appears to be less flexible with the inter-arm rotation among different subunits varying by only 2–3°. However, the “P-loop” (residues 360–370), which is likely to be involved in binding of the pyrophosphate group of AcCoA, probably moves ∼1.0 Å when the substrate binds.

### Human NAGS model

**Figure 7:**
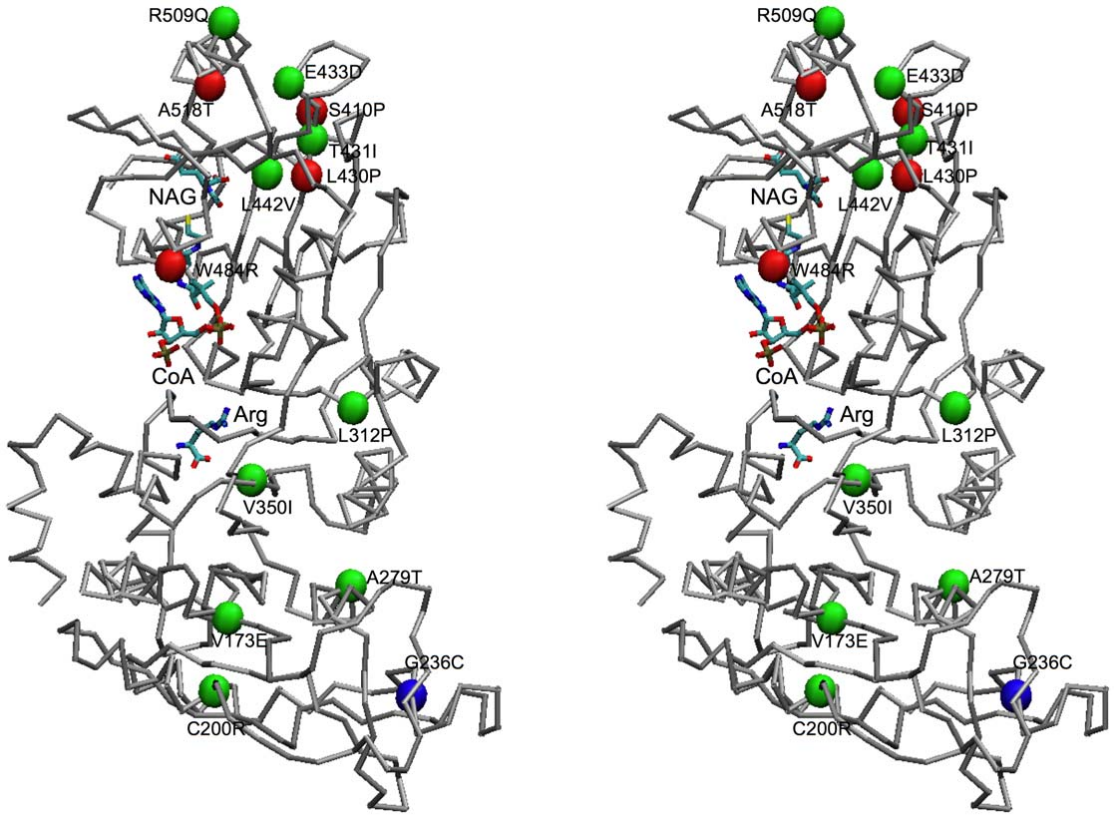
Structural model of human NAGS shown in stereo view as a Cα-trace. Modeled bound arginine, CoA and NAG are shown in stick mode. The C^α^ atoms of residues with missense mutations identified in NAGS deficient patients are shown as red (neonatal onset – severe phenotype) or green (late onset – mild phenotype) or blue (unknown onset) spheres.

Since the primary sequences of mmNAGS/K and human NAGS have ∼31% identity while the sequence identity of ngNAGS and human NAGS is only 17%, mmNAGS/K is likely to be a more reliable structural model for human NAGS than ngNAGS [5], [10]. The human NAGS structure built with mmNAGS/K as the model using Swiss-model web server [31], [32], [33] is shown in Figure 7, with naturally occurring missense mutations identified in patients with clinical hyperammonemia shown as spheres. Among 14 missense mutations, 6 are located in the AAK domain and 8 are located in the NAT domain. The four neonatal missense mutations (S410P, L430P, W484R and A518T) are all located in the NAT domain close to the putative substrate binding sites. The model predicts that the side chain of Cys200 will be close to the side chain of Cys259 and could be potentially form a disulfide bond as previously predicted [34]. It also predicts that the arginine binding site and AcCoA binding site will be close to each other, and that the orientation of the NAT domain relative to AAK domain will be intermediate between those of subunits Y and B in the mmNAGS/K structure. The model predicts that arginine binding will not cause closure of the AcCoA binding site because of the steric restraints imposed by the non-glycine hinge residue (Ala375). This prediction is consistent with arginine's role as an allosteric activator of human NAGS.

## Discussion

Although the subunit structures of bifunctional NAGS/K and classical bacterial NAGS have some similarities, there are substantial differences between them. Both have two-domain structures, consisting of a typical AAK fold and a GCN5-related NAT fold. The AAK domains are very similar, except for an extra *N*-terminal helix (Figure 3B) in mmNAGS/K. However, there are significant differences in the structures of the NAT domain, particularly in the *C*-terminal arm (Figure 3C). Importantly, the linker between the two domains consists of one amino acid in mmNAGS/K vs. three amino acids in ngNAGS, allowing much stronger interdomain interactions in mmNAGS/K than in ngNAGS, and different relative domain orientations (Figure S4A–B). As a result, the putative arginine and AcCoA binding sites are in proximity only in mmNAGS/K, creating the possibility of allosteric interactions between the binding sites.

However, the largest difference between mmNAGS/K and ngNAGS involves the quaternary structure. While the mmNAGS/K holoenzyme is organized as a tetrameric ring, ngNAGS functions as a hexamer (Figure S4C–D) [10]. The tetramer of mmNAGS is formed by AAK-AAK and NAT-NAT domain interactions which are unique to mmNAGS/K, while the hexamer of ngNAGS is stabilized by interactions between AAK domains at two major interfaces (the ecNAGK-like interface and the arginine-sensitive NAGK-like *N*-terminal interlaced helix interface) without involving the NAT domain. Arginine sensitive bacterial NAGKs that do not have a NAT domain have similar hexameric ring structures [8] confirming that a NAT domain is not important in the hexameric quaternary structure.

**Figure 8:**
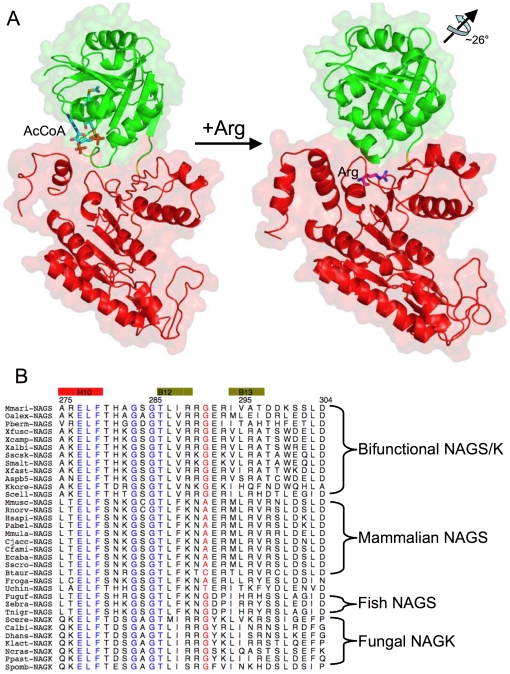
Proposed mechanism of arginine regulation of mmNAGS/K. A. The active form of mmNAGS/K with bound AcCoA (shown as a stick model) is based on the structure of subunit Y. Arginine (shown as a stick model) bound to the inactive form is based on the structure of subunit B. The NAT domain (green ribbons) rotates ∼26° relative to the AAK domain (shown as red ribbons) when arginine binds, thus preventing AcCoA from binding. The linker, Gly291, is shown in yellow. B. Sequence alignment of the arginine binding and AAK-NAT linker regions, with arginine binding amino acids in blue and linker amino acids in red.

The mechanism of NAGS activity regulation by arginine appears also to be different in the two enzyme groups. In ngNAGS, the conformational changes induced by arginine propagate from the AAK domain to the NAT domain via the interdomain linker, re-orienting the NAT domain, and as a consequence disordering the glutamate binding loops to reduce enzyme activity [5]. In contrast, in mmNAGS/K, the interdomain interaction is stronger and the marked relative domain rotation proposed to occur upon L-arginine binding would close the AcCoA binding cleft preventing AcCoA from binding. The proposed regulatory mechanism of arginine is shown in Figure 8A. These differing allosteric mechanisms for arginine in the two enzyme groups are consistent with differences in arginine titration experiments. While arginine decreases ngNAGS activity by decreasing glutamate binding affinity [5], in xcNAGS/K and mmNAGS/K, arginine binding probably prevents binding of AcCoA [1], [4].

As discussed above, and consistent with the above mechanisms, the length of the inter-domain linker appears to play a key role in the strength of the interactions between the AAK and NAT domains and the proximity of the arginine and CoA binding sites to each other. In addition, sequence comparisons indicate a clear correlation between the sequence of the linker and the allosteric effect of arginine (Figure 8B). In all bifunctional NAGS/K and fish NAGS (fugu fish, zebra fish and tetraodon) in which arginine inhibits NAGS activity, the linker consists of a glycine. When the effect of arginine is neutral, as in frogs, or activating, as in mammals, the linker contains an alanine, cysteine, or threonine, but never glycine. It appears that the steric constraints imposed by a non-glycine residue limit the magnitude of the conformational changes that can be induced by arginine and give rise to the variable allosteric effect of arginine on NAGS activity. Partial inhibition of NAGS activity by arginine in fish is further influenced by neighboring amino acid residues [4]. Even though the differences in the linker appears to play major role in the variable arginine effect on NAGS activity, other residues such as Arg276, His281, Glu366 which are located in NAT and AAK interface may also be involved.

Twenty one deleterious mutations that cause human disease (hyperammonemia) have been reported to date [34]. Among them 14 are missense substitutions that may have structural relevance. Since patients with NAGS deficiency can be rescued by the administration of the NAG analogue, *N*-carbamylglutamate, identifying deleterious mutations that are likely to produce hyperammonemia is useful clinically [35], [36]. It is therefore important to be able to predict the functional effects of amino acid substitutions on structure and function of the enzyme. In this regard a reliable protein structure of mammalian NAGS would be very useful. Unfortunately, mammalian NAGS has proven recalcitrant to crystallization and the current mmNAGS/K structure provides the most reliable model to date for human NAGS. This model allows the potential impact of NAGS mutations on structure and function to be examined.

Known naturally occurring amino acid substitutions predicted from mutations in the NAGS gene have been mapped onto a human NAGS model (Figure 7) based on the mmNAGS/K structure [34]. Since the active site is located within the NAT domain, while the AAK domain has only structural and regulatory roles, with arginine enhancing NAGS activity in human NAGS, mutations in the NAT domain might be expected to have more severe functional and clinical consequences, presenting clinically at birth or shortly thereafter, while mutations in the AAK domain might be expected to allow residual enzymatic activity and to have milder phenotypes. Indeed, 5 of the 6 mutations in the AAK domain are associated with a milder (late onset) phenotype, while the age of onset of the patient with the sixth mutation (G236C) is unknown. In contrast, 4 of the 8 missense mutations in the NAT domain are associated with a severe (neonatal onset) phenotype.

## Materials and Methods

### Cloning and protein expression and purificatio*n*

The *argA/B* gene was PCR amplified from *M. maris* MCS10 genomic DNA (generously donated by Dr. Craig Stephens, Biology Department, Santa Clara University, 500 El Camino Real, Santa Clara, CA) using Phusion™ polymerase (Finnzymes, New England BioLabs) and the primers 5′-CATATGAATCCGAATGCACCGGG-3′ and 5′-GGATCCTCATTGCGGCGCCTCAAGGGT-3′. The PCR product was cloned into a TOPO vector using a Zero-Blunt TOPO cloning kit (Invitrogen). *NdeI* and *XhoI* (New England BioLabs) were used to transfer the *argA/B* gene from TOPO to the expression vector pET28a (Novagen) using T4 DNA ligase (Invitrogen) which was then transformed into Rosetta 2 cells (Novagen) for expression. An ABI PRISM 3130 Genetic Analyzer (Applied Biosystems) was used to confirm the sequence by DNA sequencing. Protein overexpression was induced by incubating overnight with 0.2 mM IPTG at room temperature (∼298 K). The expressed protein has 20 non-native amino acid residues (MGSSHHHHHHSSGLVPRGSH) at its *N*-terminal including six His residues and a thrombin recognition site (underlined). The cells were harvested by centrifugation, suspended in 30 ml of Ni-affinity lysis buffer (20 mM NaH_2_PO_4_, 300 mM NaCl, 10% (v/v) glycerol, 10 mM β-mercaptoethanol, pH 7.4) and disrupted by sonication. The protein was then purified using an ÄKTA FPLC system (GE Healthcare) following the protocol described previously [2]. Protein concentrations were determined by the Bradford method using the BioRad protein-assay dye reagent with bovine serum albumin as a standard [37].

The Se-Met substituted NAGS/K protein was prepared using the Overnight Express Auto-induction System 2 (Novagen) as described previously [2]. In brief, the expression plasmid was transformed into a *metE* minus host strain B834(DE3) (Novagen). The clone was inoculated into 1L sterile deionized water supplemented with the chemicals provided in the kit, 125 mg L-selenomethionine and 50 mg kanamycin. Vitamin B12 (cyanocobalamin) was added to a final concentration of 100 nM and the culture was incubated at 303 K for 16 hours. After reaching stationary phase, the cells were harvested and the protein was purified as described above for the native protein.

The Se-Met substituted protein was characterized using a 4700 ABI TOF/TOF mass spectrometer (Applied Biosystem) operated in reflection positive ion mode as described previously [38]. Approximately 10 µg of purified native or Se-Met substituted protein were digested overnight at 310 K using Promega sequencing grade trypsin (enzyme/protein ratio, 1∶50, w/w) in 50 mM ammonium bicarbonate (pH 7.4). After desalting, 0.3 µL of the resulting peptide solution was mixed with 0.3 µL of saturated α-hydroxycinnamic acid and spotted on the MALDI plate. The Se-Met substituted peptides were identified using the characteristic features of the isotopic distribution of selenium. The intensity was compared to the native peptide signal at a position of −57 Da to establish that more than 80% of the protein was Se-Met substituted.

### Site-directed mutagenesis

Site-directed mutant genes of *M. maris* NAGS were created by utilizing primers containing the desired mutations (Table 2) and Quik Change Mutagenesis Kit according to the manufacturer's protocol (Strategene). Initially, a thermal cycle was applied to denature double-stranded *M. maris* NAGS/K wild-type plasmid, and then the appropriate mutagenic primer was annealed to it. *PfuTurbo* DNA polymerase from the Quik Change kit was used to extend the primer without primer displacement and to seal nicks. The product was treated with *Dpn*I to digest parental plasmids, which are susceptible because of their methylated DNA. Then, transformation into XL10-Gold ultracompetent cells allowed conversion of mutated single stranded DNA to double stranded plasmid DNA. Finally, the sequences of mutant DNA sequences were verified by an ABI PRISM 3130 Genetic Analyzer (Applied Biosystems) using the commercially available primer pair annealed to the plasmid promoter and terminator regions.

**Table 2:** Mutagenesis primers.

Mutants	Primers
I106M	ACCCGCGACGAGGCG**ATG**CCGATCATCCGGGATAC
I294M	CAGGGGCGAGCGG**ATG**GTCGCCACCGATGAC
L376M	ACGGTGTGGAACCGG**ATG**GTCGATTATTGCAC

To increase the number of methionine residues available for experimental phasing, Ile106, Ile294 and L376 were mutated to Met simultaneously, using the sequences of homologue protein from other bacteria as a guide. The mutant was overexpressed and purified in the same way as wild-type protein.

### Biochemical characterization: Activity assays and cross-linking experiments

The NAGS and NAGK activities of mmNAGS/K were measured in the presence of different concentrations of L-arginine using the method described previously [1]. In NAGS assays, 0.16 µg of enzyme were incubated in 100 µl of assay solution containing 2.5 mM AcCoA, 10 mM L-glutamate and 50 mM Tris-HCl pH 8.5 at 293 K for 5 min. The reaction was stopped with 100 µl of 30% TCA. NAG was quantified using liquid chromatography-mass spectrometry. The arginine titration curve was obtained using different concentration of L-arginine. For NAGK activity, the enzyme (0.2 µg) was incubated in the 500 µl assay buffer containing 20 mM ATP, 100 mM NAG, 100 mM NaCl, 40 mM MgCl_2_, 400 mM hydroxylamine and 20 mM Tris-HCl pH 7.4 for 30 min at 310 K. The reaction was terminated by adding 450 µl ferric chloride solution (5% FeCl_3_, 5% TCA and 0.3 M HCl). The absorption for the colored reaction mixture was measured at 540 nm. Cross-linking experiments were performed using the protocol described by Davies and Stark [39]. mmNAGS/K and xcNAGS/K (0.15 µg) were incubated with cross-linking reagent dimethyl suberimidate (0.25 µg) in 50 µl solution containing 200 mM triethanolamine, pH 8.25 three hours at 298 K. Samples with and without cross-linking reagent were subjected to sodium dodecyl sulfate polyacrylamide get electrophoresis (NuPAGE 4–12% Bis-Tris gel) in MES SDS buffer (50 mM MES, 50 mM Tris base, 0.1% SDS, 1 mM EDTA, pH 7.3) and stained with Coomassie blue. Benchmark with premixed different molecular weights of protein standards was purchased from Invitrogen.

### Crystallization

The purified protein was concentrated to 16 mg/ml with an Amicon-Y30 membrane concentrator (Millipore). Screening for crystallization conditions was performed using sitting-drop vapor diffusion in 96-well plates (Hampton Research) at 291 K by mixing 2 µl of the protein solution with 2 µl of the reagent solution from the sparse matrix Crystal Screens 1 and 2, and Index screen (Hampton Research). Further optimizations of the crystallization conditions were carried out using the hanging-drop vapor diffusion method.

Before crystallization, the enzyme was incubated with 25 mM CoA and 100 mM glutamate at 277 K for 1 hour. Different crystallization conditions yielded several different crystal forms: orthorhombic (space group P2_1_2_1_2_1_), trigonal (space group P3_1_21) and hexagonal (space group P6_2_22). The best orthorhombic form crystals for native and NAGS were grown from a well solution containing 25% PEG3350, 200 mM NaCl and 100 mM Bis-Tris, pH 6.5. The best crystallization conditions for the Se-Met substituted mutant were 25% PEG3350, 200 mM sodium malonate, pH 7.0. The trigonal form crystals were obtained from a well solution containing 23% PEG400, 100 mM Bis-Tris pH 6.5 and 1 mM 5-amino-2,4,6-triiodoisophthalic acid. Hexagonal form crystals were produced from a solution containing 25% PEG3350, 100 mM Li_2_SO_4_ and 100 mM Tris pH 9.0 and have the same morphology as the hexagonal bipyramid crystals of xcNAGS/K [2].

### Data collection and structure determination

Before data collection, crystals were transferred from the cover slip on which they were grown to a well solution supplemented with 25% ethylene glycol and then frozen by direct immersion into liquid nitrogen. Data sets for Se-Met substituted proteins were collected at the selenium adsorption edge, the inflection point and a remote position at the SER-CAT advanced Photon Source. Data sets for the native crystals were collected to ∼3.1 Å resolution for the orthohombic crystal form and ∼4.3 Å resolution for the trigonal crystal form, respectively. All data were processed using the HKL2000 package [40]; statistics are summarized in Table 1. The diffraction data for the hexagonal crystal form of xcNAGS/K were reported previously [2].

The mmNAGS structure was solved using the three wavelength MAD (3W-MAD) protocol of Auto-Rickshaw: the EMBL-Hamburg automated crystal structure determination platform [41]. The input diffraction data were prepared and converted for use in Auto-Rickshaw using programs in the CCP4 suite [42]. The structure-factor amplitudes of anomalous scatterers (*F_A_* values) were calculated with the program SHELXC [43]. Based on an initial analysis of the data, the maximum resolution for substructure determination and initial phase calculation was set to 3.2 Å. The 25 Se atoms were identified using the program SHELXD [44] and the correct hand for the substructure was determined using the programs ABS [45] and SHELXE [43]. Occupancies of all substructure atoms were refined and initial phases were calculated with MLPHARE [42]. Density modification, phase extension and NCS-averaging were performed with RESOLVE [46]. A partial α-helical model contained 1503 residues out of a total of 1764 was produced with HELICAP [47]. After model adjustments with Coot [48], structural refinements were performed with Phenix [49]. During the initial stages of the refinement, NCS restraints were used and *R* and *R*_free_ dropped to 32.0 and 42.6%, respectively, but did not decline further. In subsequent refinements, NCS restraints were removed and the structural models for each subunit were adjusted individually, revealing significant conformational differences between subunits. The final refinement without NCS restraints, but with translation/libration/screw parameters [50] included resulted in *R* and *R*_free_ values of 18.9% and 25.6%, respectively. The rather large difference between *R* and *R*_free_ is probably due to anistropic diffraction. The translation/libration/screw groups were selected based on five structural regions per subunit as shown in Figure 1A. The final model contains 4 protein subunits, 1 CoA, 2 malonates, 2 ethylene glycols, 1 sulfate group and 84 water molecules. The native structure was refined and modeled in the same way as the Se-Met substituted protein, but with a new set of random reflections chosen for the calculation of *R*_free_. The final native structure model has 4 protein subunits, 2 CoAs, 2 glutamates and 6 water molecules. Refinement statistics for the final refined model are given in Table 1.

The structure of the trigonal crystal form was solved in space group P3_1_21 with the dimer of subunits A and X of the orthorhombic crystal form as the search model and the program Phaser [51], [52]. Rigid body refinement brought *R* and *R*_free_ values to 42.9% and 43.1%, respectively, confirming the correctness of the structural solution. Further refinement with the reference model (Se-Met substituted mmNAGS/K structure) restraints resulted in *R* and *R*_free_ values of 27.4% and 41.9%, respectively (Table S1). The structural refinement greatly improved when additional restraints from known homologous structures were introduced [53].

The structure of the hexagonal form of xcNAGS/K was solved in space group P6_2_22 using CaspR, the web-server for automatic molecular replacement [54] and the structure of mmNAGS/K as the search model. The solution from molecular replacement is consistent with the electron density map constructed using experimental phases from MAD data [2]. Rigid body refinement reduced *R* and *R*_free_ values to 49.6% and 48.7%, respectively. After mutating residues from the mmNAGS/K sequence to the xcNAGS/K sequence and manual model rebuilding, further refinement brought *R* and *R*_free_ values down to 31.9% and 38.4%, respectively. Since the dataset was collected at the selenium edge from Se-Met substituted crystals and contained anomalous signals, scattering factors were included in the refinement. The refinement improved significantly for low resolution data with the inclusion of anomalous diffraction data, as reported [55]. Data collection and final refinement statistics for the trigonal crystal form of mmNAGS/K and hexagonal form of xcNAGS/K are listed in Table S1.

### Structural modeling

The structural model for human NAGS subunit was built using the Swiss-Model web server and the mmNAGS/K structure (subunit A) as the template [31], [32], [33]. The model, checked using program PROCHECK [56], has good stereo geometry with 86.2% dihedral angles in the most favored region of the Ramachandran plot and 11.9% in the generally allowed region. There are 9 bad contacts in the model. Coordinates for human NAGS model are provided in Supporting Information S1 (hNAGS-model.pdb).

Figures were drawn using programs Alscript [57], Pymol [58] and VMD [59]. The secondary structure was assigned using STRIDE web server [60].

### Protein Data Bank accession numbers

The final refined coordinates of the native and Se-Met substituted mutant structures of mmNAGS/K in orthorhombic space group P2_1_2_1_2_1_ and in trigonal space group P3_1_21, and the Se-Met substituted structure of xcNAGS/K in hexagonal space group P6_2_22 have been deposited in RCSB Protein Data Bank with accession codes, 3S6H, 3S6G, 3S7Y and 3S6K, respectively.

## Supporting Information

Figure S1 A. Molecular packing of mmNAGS in the unit cell in space groups P2_1_2_1_2_1_ and P3_1_21, and of xcNAGS/K in space group P6_2_22. Different tetramers are shown in different colors. B. Ribbon diagram of native mmNAGS/K tetramer structure with subunit A (red), subunit B (green), subunit X (purple -gray) and subunit Y (yellow). Two bound CoA and glutamate molecules are shown as space-filling models. Glutamate binding site is remote from the non-functional CoA binding site.
(TIF)

Figure S2 A. Stereo diagram of the superimposition of the AAK domain of mmNAGS/K (shown as red ribbon) and ecNAGK (shown as pink ribbon) (PDB 1GS5). AMPPNP (ATP analog) and NAG are shown as light-blue sticks. The side chains of key catalytic residues are shown as yellow sticks. B. Stereo diagram of superimposition of AAK domain of mmNAGS/K (red ribbon) and arginine bound ngNAGS (pink ribbon, PDB 3D2P) showing the proposed arginine binding site. Arginine (shown in light-blue sticks) is located in the cavity formed by the loop connecting helix H10 and β strand B12. Side chains of key site residues are shown in yellow sticks. C. Stereo diagram of superimposition of the NAT domain of mmNAGS/K (red ribbon) and ngNAGS (pink ribbon, PDB 3B8G) showing the proposed CoA (yellow sticks) binding site in the V-shaped cleft formed by the *N*- and *C*-terminal arms of NAT domain. Side chains of key site residues are shown as light-blue sticks.
(TIF)

Figure S3 A. Relative rotation of the AAK and NAT domains of the four subunits of unliganded mmNAGS/K. Stereo view of the Cα-trace representation of the four subunits of the asymmetric unit with the core β-sheets of the AAK domains superimposed. Red, subunit A; green, subunit B; purple-grey, subunit X; yellow, subunit Y. B. Ribbon diagram of subunit B with modeled CoA, NAG and arginine bound. The circle indicates the proposed steric clash between CoA and the arginine-binding loop in the conformation of subunit B. CoA, NAG and arginine are shown in sticks. C. Ribbon diagram of subunit Y with modeled CoA, NAG and arginine bound. The coordinates of CoA and NAG were obtained by structurally superimposing the NAT domain of ngNAGS (PDB 3B8G) and that of subunit B or subunit Y of mmNAGS/K.
(TIF)

Figure S4 Comparison of mmNAGS/K and ngNAGS. A. Stereo diagram of superimposition of mmNAGS/K (subunit A, red ribbon) and arginine bound ngNAGS (pink ribbon, PDB 3D2P) with the core β-sheets of the AAK domains superimposed. Bound CoA and arginine are shown in space-filling mode. B. Stereo diagram of superimposition of mmNAGS/K (subunit A, red ribbon) and CoA and NAG bound ngNAGS (pink ribbon, PDB 3B8G) with the core β-sheets of the AAK domains superimposed. Bound CoA (light-blue) and NAG (green) are shown in space-filling mode. C. Simplified structural model of the mmNAGS/K tetramer. D. Simplified structural model of the ngNAGS hexamer (PDB 3B8G). Different subunits are shown in different colors.
(TIF)

Table S1 Diffraction data and refinement statistics for mmNAGS/K and xcNAGS/K in space groups P3_1_21 and P6_2_22.
(DOC)

Supporting Information S1 (PDB)
