# Supplementary figures and images for: A Novel N-Acetylglutamate Synthase Architecture Revealed by the Crystal Structure of the Bifunctional Enzyme from Maricaulis maris
Source: PLoS One. 2011 Dec 12;6(12):e28825. doi: 10.1371/journal.pone.0028825 (PMC3236213; doi:10.1371/journal.pone.0028825)

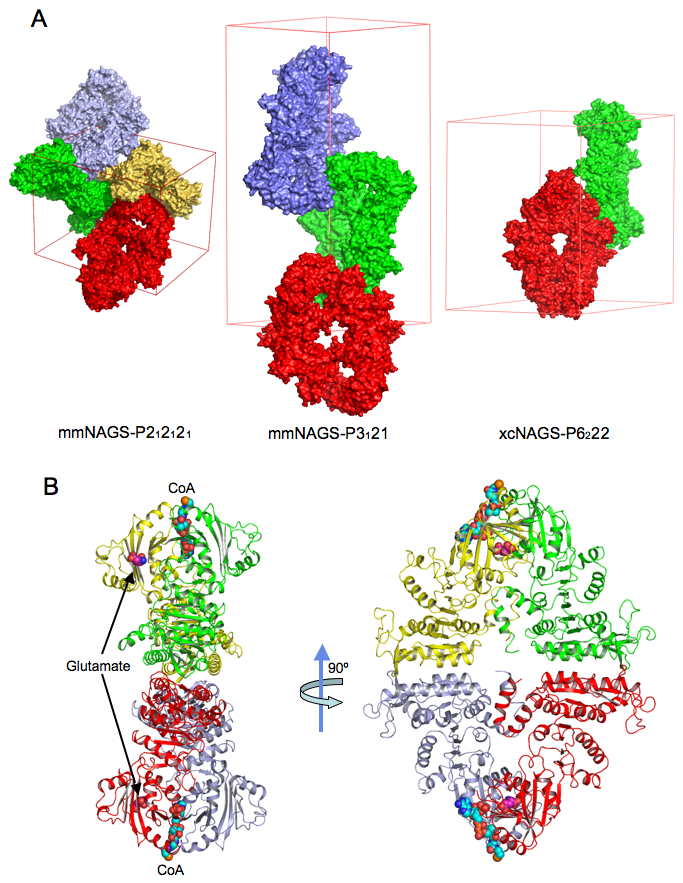

Supplement: Figure S1 — A. Molecular packing of mmNAGS in the unit cell in space groups P212121 and P3121, and of xcNAGS/K in space group P6222. Different tetramers are shown in different colors. B. Ribbon diagram of native mmNAGS/K tetramer structure with subunit A (red), subunit B (green), subunit X (purple -gray) and subunit Y (yellow). Two bound CoA and glutamate molecules are shown as space-filling models. Glutamate binding site is remote from the non-functional CoA binding site. (TIF) [file pone.0028825.s001.tif]

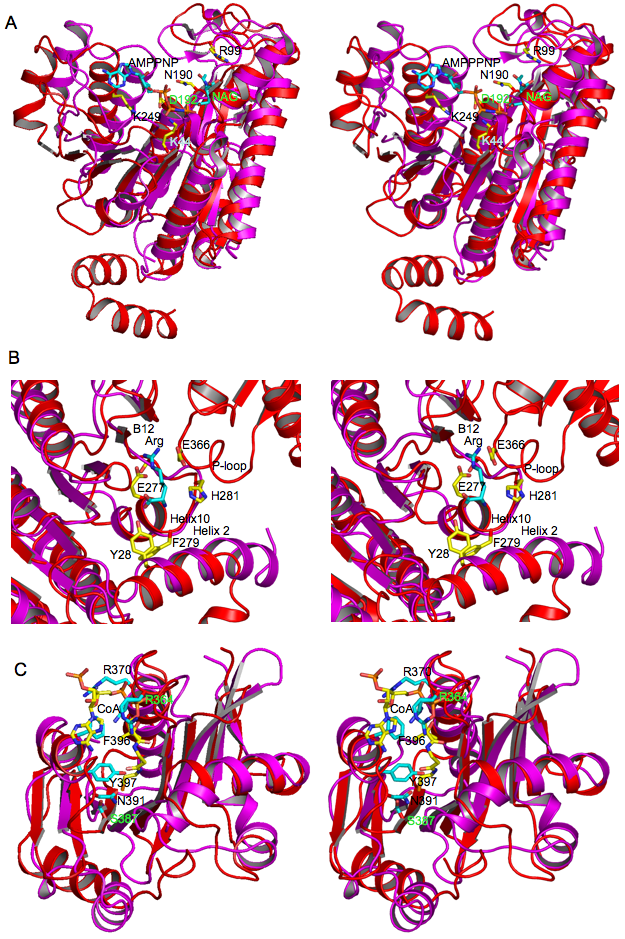

Supplement: Figure S2 — A. Stereo diagram of the superimposition of the AAK domain of mmNAGS/K (shown as red ribbon) and ecNAGK (shown as pink ribbon) (PDB 1GS5). AMPPNP (ATP analog) and NAG are shown as light-blue sticks. The side chains of key catalytic residues are shown as yellow sticks. B. Stereo diagram of superimposition of AAK domain of mmNAGS/K (red ribbon) and arginine bound ngNAGS (pink ribbon, PDB 3D2P) showing the proposed arginine binding site. Arginine (shown in light-blue sticks) is located in the cavity formed by the loop connecting helix H10 and β strand B12. Side chains of key site residues are shown in yellow sticks. C. Stereo diagram of superimposition of the NAT domain of mmNAGS/K (red ribbon) and ngNAGS (pink ribbon, PDB 3B8G) showing the proposed CoA (yellow sticks) binding site in the V-shaped cleft formed by the N- and C-terminal arms of NAT domain. Side chains of key site residues are shown as light-blue sticks. (TIF) [file pone.0028825.s002.tif]

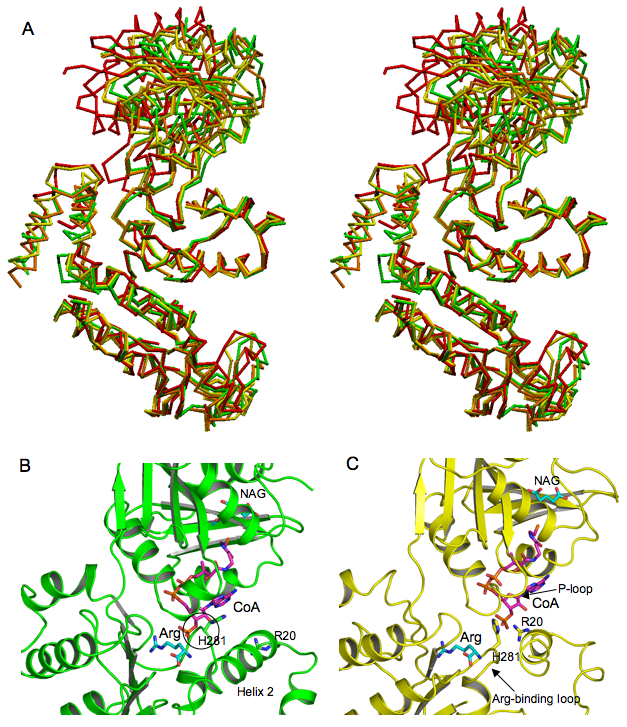

Supplement: Figure S3 — A. Relative rotation of the AAK and NAT domains of the four subunits of unliganded mmNAGS/K. Stereo view of the Cα-trace representation of the four subunits of the asymmetric unit with the core β-sheets of the AAK domains superimposed. Red, subunit A; green, subunit B; purple-grey, subunit X; yellow, subunit Y. B. Ribbon diagram of subunit B with modeled CoA, NAG and arginine bound. The circle indicates the proposed steric clash between CoA and the arginine-binding loop in the conformation of subunit B. CoA, NAG and arginine are shown in sticks. C. Ribbon diagram of subunit Y with modeled CoA, NAG and arginine bound. The coordinates of CoA and NAG were obtained by structurally superimposing the NAT domain of ngNAGS (PDB 3B8G) and that of subunit B or subunit Y of mmNAGS/K. (TIF) [file pone.0028825.s003.tif]

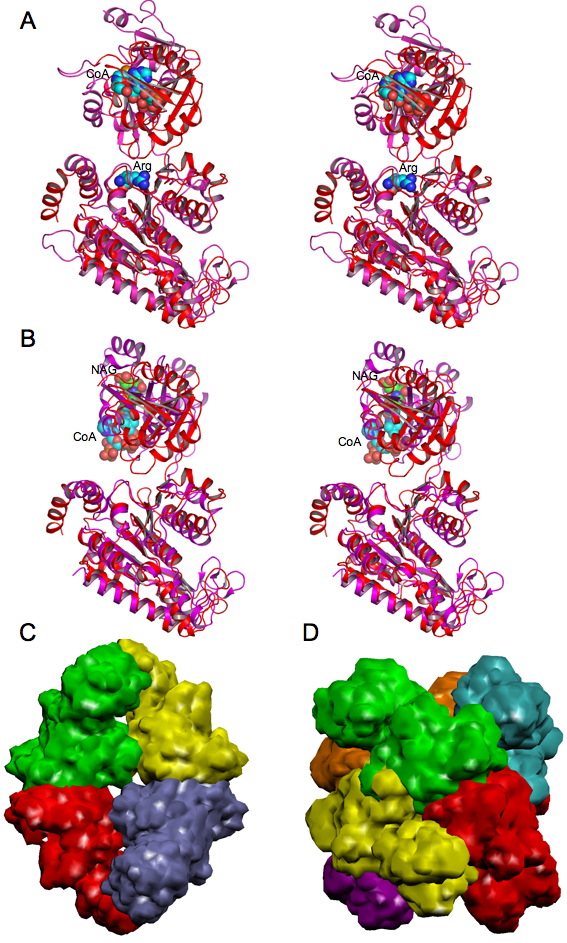

Supplement: Figure S4 — Comparison of mmNAGS/K and ngNAGS. A. Stereo diagram of superimposition of mmNAGS/K (subunit A, red ribbon) and arginine bound ngNAGS (pink ribbon, PDB 3D2P) with the core β-sheets of the AAK domains superimposed. Bound CoA and arginine are shown in space-filling mode. B. Stereo diagram of superimposition of mmNAGS/K (subunit A, red ribbon) and CoA and NAG bound ngNAGS (pink ribbon, PDB 3B8G) with the core β-sheets of the AAK domains superimposed. Bound CoA (light-blue) and NAG (green) are shown in space-filling mode. C. Simplified structural model of the mmNAGS/K tetramer. D. Simplified structural model of the ngNAGS hexamer (PDB 3B8G). Different subunits are shown in different colors. (TIF) [file pone.0028825.s004.tif]
