# Supplementary material for: A Novel N-Acetylglutamate Synthase Architecture Revealed by the Crystal Structure of the Bifunctional Enzyme from Maricaulis maris
Source: PLoS One. 2011 Dec 12;6(12):e28825. doi: 10.1371/journal.pone.0028825 (PMC3236213; doi:10.1371/journal.pone.0028825)
Supplement: Table S1 — Diffraction data and refinement statistics for mmNAGS/K and xcNAGS/K in space groups P3121 and P6222. (DOC) [file pone.0028825.s005.doc]

**Table S1.** Diffraction data and refinement statistics for mmNAGS/K and xcNAGS/K in space groups P3_1_21 and P6_2_22

|  | mmNAGS/K | | xcNAGS/K |
| --- | --- | --- | --- |
| Data |  |  |  |
| Morphology | Rhombohedron | Hexagonal bipyramid | Hexagonal bipyramid |
| Space group | P3_1_21 | P6_2_22 | P6_2_22 |
| Resolution (Å) | 50.0-4.3 | <6.0 | 50.0-2.8 |
| Highest resolution shell (Å) | 4.45-4.3 |  | 2.91-2.80 |
| Space group | P3_1_21 | P6_2_22 | P6_2_22 |
| Unit-cell parameters (Å) | *a* = 95.1 | *a* = 134.6 | *a* = 133.2 |
|  | *b* = 95.1 | *b* = 134.6 | *b* = 133.2 |
|  | *c* = 253.0 | *c* = 192.1 | *c* = 191.4 |
| No. of ASU | 2 | 1 | 1 |
| Matthew’s coefficient | 2.1 | 5.2 | 4.8 |
| Solvent (%) | 41.9 | 76.3 | 74.0 |
| Measurements | 58,162 |  | 362,784 |
| Unique reflections | 9,506(913) |  | 21,146 (1,336) |
| Redundancy | 6.1(5.6) |  | 17.2 (8.0) |
| Completeness (%) | 99.6(99.5) |  | 83.3 (54.0) |
| *R*_merg_ ^b^ | 0.055(0.915) |  | 0.065 (0.334) |
| <*I/σ*(*I*)> | 23.3(1.9) |  | 55.1(2.5) |
| Refinement |  |  |  |
| Reflections, working set | 8,736 |  | 33,401 |
| Reflections, test set | 867 |  | 1,690 |
| Total atom (non-H) | 6,683 |  | 3,407 |
| Protein atoms | 6,683 |  | 3,407 |
| Water atoms | 0 |  | 0 |
| *R* | 0.274 (0.452) |  | 0.321 (0.412) |
| *R*_free_ | 0.419 (0.518) |  | 0.388 (0.470) |
| Rmsd bond lengths (Å) | 0.015 |  | 0.010 |
| Rmsd bond angles (º) | 2.456 |  | 1.647 |
| Ramachandran plot (%) |  |  |  |
| Favored | 78.9 |  | 65.5 |
| Allowed | 18.4 |  | 28.7 |
| Generous | 1.5 |  | 5.1 |
| Disallowed | 1.2 |  | 1.3 |

^a^ Figures in brackets apply to the highest-resolution shell.

^b^ *R*_merg_ = Σ*_h_*Σ*_i_**I*(*h*,*i*)*-<I*(*h*)*>*/Σ*_h_*Σ*_i_I*(*h*,*i*), where *I*(*h*,*i*) is the intensity of the *i*th observation of reflection *h*, and < *I*(*h*)> is the average intensity of redundant measurements of reflection *h*.
